# An Accurate Altimetry Method for High-Altitude Airburst Fuze Based on Two-Dimensional Joint Extension Characteristics

**DOI:** 10.3390/s25072329

**Published:** 2025-04-06

**Authors:** Liwen Pan, Yao Zhang, Qianyu Wang, Shuhuan He, Xi Pan

**Affiliations:** School of Mechatronical Engineering, Beijing Institute of Technology, Beijing 100081, China; bit_panlw@126.com (L.P.); bit_zhangy@126.com (Y.Z.); 13671078750@163.com (Q.W.); bit_hesh@126.com (S.H.)

**Keywords:** high-altitude airburst fuzes, improved constant false alarm rate, extension characteristic, feature region, altimetry method

## Abstract

Considering the challenge of precise altimetry for high-altitude airburst fuzes, this paper proposes a two-dimensional joint extension characteristic altimetry method based on an improved constant false alarm rate (CFAR) detection and an accurate feature region extraction approach. First, an improved CFAR detection method with secondary protection windows is introduced to effectively mitigate the masking effect caused by conventional CFAR algorithms. The fuze-to-ground distance-based height measurement is achieved by leveraging the geometric relationship between the maximum and minimum slant distances and the impact angle. Then, to enhance altimetry accuracy under low signal-to-noise ratio (SNR) conditions, a 2D joint accurate altimetry approach is implemented by integrating Doppler-dimension extension characteristics with the conventional range-based method. The estimated impact angle is further refined using the proposed feature region extraction method. The final results demonstrate that for high-altitude airburst fuzes operating at burst altitudes between 70 m and 100 m, the proposed 2D joint altimetry algorithm provides more accurate and robust distance measurements. Under an SNR of −10 dB, the root mean square error (RMSE) is less than 2.38 m, with an error rate of approximately 3%. Notably, even at an SNR of −15 dB, the RMSE remains below 4.76 m, with an error rate not exceeding 5%, highlighting the robustness of the proposed method under low-SNR conditions.

## 1. Introduction

A fuze is a control system that utilizes environmental, target, or platform information to precisely control the detonation of ammunition according to a predetermined strategy while ensuring both operational and launch safety [1,2]. As the operational range of fuzes increases, weapon systems can detect targets at greater distances for detonation, thereby enhancing hit accuracy and lethality. Common fuzes, categorized by their operational range from near to far, include contact fuzes with an activation distance of 0 m, laser and infrared fuzes with an effective range of approximately 1–10 m, and radio proximity fuzes operating within a range of 10–30 m. To meet the demands of modern warfare, the concept of high-altitude airburst fuzes with an extended operational range of 60–120 m has been proposed. However, as the fuze detonation range increases, the challenges associated with signal strength and ranging accuracy also intensify. Research on long-range detection, such as drone detection using frequency-modulated continuous wave (FMCW) radar, demonstrates the feasibility of detecting distant targets with high accuracy, which is relevant for high-altitude airburst fuzes [3]. Consequently, research on fuzes with an operational range exceeding 30 m remains limited. Therefore, investigating high-altitude airburst fuzes is of significant practical relevance. Due to the constraints of volume, cost, and power consumption, the transmit power and antenna gain of high-altitude and high-frequency fuze are limited, which leads to low power of the received target echo signal, poor SNR, and difficulty in extracting target range and azimuth information [4]. At the same time, in order to improve the SNR of the fuze echo signal, a narrow-beam antenna with higher gain is usually used for detection, but the angular coverage of the narrow-beam antenna is limited, and when the ground incidence angle of the center of the antenna beam is large, the minimum slant distance that can be measured by the fuzes is larger than the error between the actual height. Time-domain ranging algorithms have been proposed to enhance FMCW sensor performance, achieving high precision while maintaining low computational complexity [5]. References [6,7,8] show that some other types of radar use the barycenter estimation method, the time delay characteristic method, and the three-beam antenna joint ranging method to estimate the incidence angle. Infrared image sensors have also been explored for small-target detection in trajectory correction fuzes, demonstrating strong anti-noise performance and effective clutter suppression [9].

Shi et al. [10] established a limit sensitivity model for high-altitude airburst radio fuzes under the condition of vertical irradiation by a narrow-beam antenna on the ground. It highlights the necessity for high-altitude airburst radio fuzes to fully utilize the characteristics of both the echo signal and noise to extract signals buried in noise effectively. In reference [11,12], the authors propose a noise-based approach to design an LMS (Least Mean Squares) adaptive noise canceller for effective noise suppression. Additionally, a multivariate quadratic function is employed for dimensionality reduction, enhancing the convergence performance of the LMS adaptive algorithm. Wang et al. [13] apply empirical mode decomposition (EMD) and utilizes autocorrelation energy for soft-threshold noise reduction in processing FMCW radar signals. In [14], a noise reduction method for heart sound signals is proposed by combining complementary ensemble empirical mode decomposition (CEEMD) with wavelet packet analysis. In [15], signal separation is achieved through compressed sensing reconstruction based on wavelet decomposition. Dragomiretskiy et al. [16] propose a variational mode decomposition (VMD)-based adaptive non-recursive method for signal noise reduction. In [17], the signal is first decomposed using VMD, and then an intermittent threshold noise reduction algorithm is applied to process the decomposed noise components. In reference [18,19], the maximum value of the maximum likelihood function is determined by searching for the function’s zeros using Newton’s algorithm. A maximum likelihood estimation algorithm based on large parameter approximation is proposed. However, it has higher complexity and greater computational demands. In [20], a phase-weighted average frequency estimation algorithm based on the correlation function is introduced, while its frequency estimation range is constrained by the phase difference, resulting in a limited estimation range. Liu et al. [21] proposed a novel one-dimensional (1D) generalized morphological filtering method based on wavelet structuring elements. Chen et al. [22] addressed the issue of prevailing fault period estimation methods, which are prone to failure in the presence of strong periodic noise. In [23], signal amplitude and phase information were accumulated simultaneously, and echo energy was coherently summed after phase compensation to enhance signal accumulation gain. In reference [24], the authors introduced a two-dimensional FFT-based method for estimating target range and velocity information, optimized using a relative distance evaluation function.

In view of the limited computing resources and stringent real-time requirements of fuzes, the adaptive filtering algorithm in the aforementioned methods exhibit challenges such as high computational complexity, poor robustness, and difficulty in ensuring real-time performance. The mode decomposition method depends on the noise model to some extent, and the parameter selection is sensitive. Mode decomposition methods are somewhat dependent on the noise model and are highly sensitive to parameter selection. Meanwhile, non-coherent signal integration relies solely on the amplitude information of the echo signal, resulting in a certain degree of gain loss. Moreover, this integration loss increases as the signal-to-noise ratio decreases. Although coherent integration offers higher integration gain, its engineering implementation is more complex. Additionally, in fuze application scenarios, range migration and Doppler shift can occur, further complicating signal processing. While the two-dimensional FFT method effectively enhances the signal-to-noise ratio, it is computationally intensive and requires significant processing resources.

In this paper, a two-dimensional (2D) joint accurate altimetry algorithm based on range-Doppler joint extension characteristics is proposed to achieve precise detection for high-altitude and high-frequency fuzes. First, an altimetry algorithm based on distance extension characteristics is introduced, leveraging the geometric relationship between the maximum and minimum slant distance and the impact angle. For convenience in description and to maintain consistency with the slant distance measurement terminology, the “distance dimension” used in this paper actually refers to the “range dimension”. To enhance the adaptability of high-altitude airburst fuzes, an improved CFAR detection method combined with a clustering approach is employed. Furthermore, to mitigate height measurement errors in low SNR conditions, a 2D joint accurate altimetry algorithm is developed. This method utilizes the joint distance-Doppler extension characteristics to calculate the detectable beamwidth of the antenna, enabling accurate estimation of both the impact angle and altitude even under low SNR conditions. Compared with traditional altimetry algorithms, the proposed approach reduces computational complexity while significantly improving measurement accuracy and robustness.

## 2. Theoretical Analysis

### 2.1. Modelling of FM Fuze Ground Echo Signals

The detection target of the fuze is the ground area irradiated by the antenna. Due to the high altitude at which the high-altitude airburst fuze operates, the antenna’s irradiation range is large, and the ground area cannot be considered a single point target. Therefore, it is necessary to model the echo signal from the ground area and analyze the characteristics of the fuze’s echo signal. In this paper, the ground echo signal is obtained by coherently superimposing the echo signals from each scattering unit of the target. Additionally, the high-altitude airburst fuze utilizes a sawtooth wave linear frequency modulation (FM) signal [25]. The fixed distance expression for the sawtooth wave linear FM signal is as follows:

(1) $$R=\frac{cT_{m}}{2\Delta F}(f_{bv}+f_{d})$$

where R is the distance from the target to the radio fuze, ΔF is the modulated frequency deviation, $T_{m}$ is the period of the modulation signal, $f_{bv}$ is the frequency rate of the target’s beating frequency, and $f_{d}$ is the Doppler frequency due to relative motion, where $f_{d}=2v_{R}f_{0}/c$. Here, $v_{R}$ is the relative velocity between the fuze and the target, $f_{0}$ is the center frequency, and *c* is the speed of light.

Considering the ballistic environment in which high-altitude airburst fuzes operates, the geometric model of the ground echo region in this paper is based on the following three assumptions:

1. It is assumed that the overall shape of the ground is approximated as a flat, rough surface, with the height fluctuations of the rough surface being much smaller than the distance from the fuze to the ground;
2. It is assumed that the E-plane and the H-plane of the main beam of the antenna have the same beamwidth. Here, the E-plane and H-plane are two important planes used to describe the radiation direction and amplitude of the antenna. The main beam of the fuze antenna is approximated as a cone, with the center of the antenna’s main beam located along the axis of the cone and the beamwidth being the cone’s angle;
3. It is assumed that the direction of the antenna’s main beam center does not coincide with the tangent direction of the trajectory but rather forms a certain angle with it. Based on practical engineering installation requirements, the fuze is typically mounted on the side or rear of the weapon’s warhead, and the antenna’s beam direction forms an approximate 35° angle with the trajectory’s tangent.

**Figure 1:**
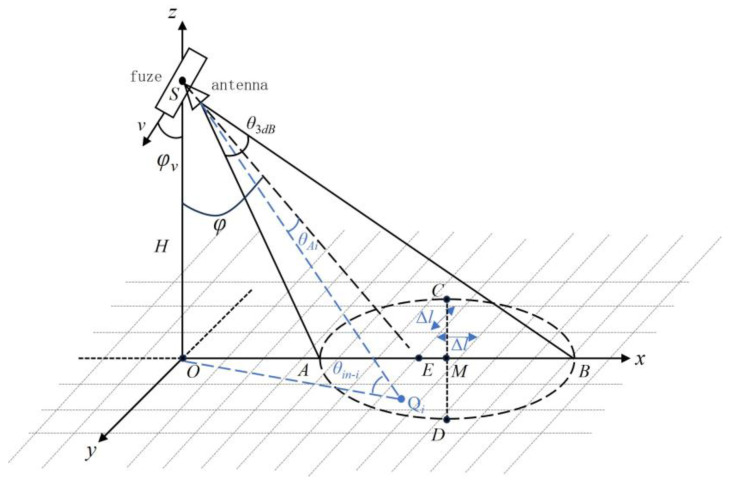
Geometric model of the fuzed ground echo region.

As shown in Figure 1, at a certain moment, the fuze is located at point S on the *z*-axis, moving within the XOZ plane with the vertical height H from the ground. The beam’s center ground incidence angle is φ, the fuze’s velocity is v, the fuze’s velocity ground incidence angle is $\varphi_{v}$, and $\theta_{3dB}$ represents the 3 dB beamwidth of the antenna. According to Assumption 3, it can be inferred that the high-altitude airburst fuze’s antenna beam generally irradiates the ground at a downward angle, so the shape of the ground area irradiated by the antenna beam is elliptical. According to the geometric relationship shown in Figure 1, the coordinates of the intersection point E of the antenna’s main beam with the ground are (*Htan*(*φ*), 0, 0), from which the unit vector in the direction of the antenna’s main beam center denoted as $\vec{e}$ can be obtained. Additionally, using *H*, *φ*, and $\theta_{3dB}$, the coordinates of the four vertices and the center point of the elliptical echo region on the ground can be calculated.

Furthermore, let the coordinates of a point $Q_{i}$ inside the elliptical region irradiated by the antenna beam be (*x_i_*, *y_i_*, 0). Based on the vector $\vec{SQ_{i}}$ from the fuze antenna at point *S* to $Q_{i}$, which lies within the main beam region, the coordinates of $Q_{i}$ must satisfy the following constraint conditions:

(2) $$\theta_{Ai}=<{\vec{SQ}}_{i},\vec{e}>=\arccos\left[\frac{x_{i}\sin(\varphi )+H\cos(\varphi )}{\sqrt{{x_{i}}^{2}+y_{i}^{2}+H^{2}}}\right]\leq \frac{\theta_{3dB}}{2}$$

To obtain the echo generated by the ground echo region, this paper employs a square uniform segmentation method to decompose the region into many uniform and independent grid-like scattering units. Each scattering unit is regarded as a point target, and the echo signal from the ground echo region is obtained by summing the echo signal from each scattering unit.

If $Q_{i}$ is the center point of a square scattering unit with side length Δl within the elliptical echo region irradiated by the antenna beam, and a Gaussian function model is used to approximate the antenna, the corresponding antenna gain for this scattering unit is given by

(3) $$G_{i}=\exp\left(-1.4{\theta_{Ai}}^{2}/\theta_{3\mathrm{dB}}^{2}\right)$$

The incident angle $\theta_{in-i}$ of the electromagnetic wave for this scattering unit is the angle between the vector from the point $Q_{i}$ to the fuze antenna *S* and the unit normal vector $\vec{q}=(0,0,1)$ of the square scattering unit:

(4) $$\theta_{in-i}=<{\vec{\mathrm{SQ}}}_{i},\vec{q}>=\arccos\left[\frac{H}{\sqrt{{x_{i}}^{2}+y_{i}^{2}+H^{2}}}\right]$$

Based on the geometric model of the ground echo region and the point target echo signal model, the echo signal $s_{ri}(t)$ corresponding to the *i*-th scattering unit can be obtained. By coherently summing the echo signals of all scattering units, the fuze ground echo signal $s_{r}(t)$ can be derived as follows:

(5) $$s_{r}(t)=\sum_{i=1}^{N_{u}}\sqrt{\frac{2P_{t}{G_{i}}^{2}\lambda^{2}\sigma_{i}}{{\left(4\pi \right)}^{3}R_{i}{\left(t\right)}^{4}}}\cos\left\{2\pi \left[f_{0}\left(t-\frac{2R_{i}(t)}{c}\right)+\left.\left.\frac{1}{2}\beta {\left(t-\frac{2R_{i}(t)}{c}\right)}^{2}\right]+\phi_{0}+\varphi_{0}\right\}\right.\right.$$

Similarly, the expression for the beating signal $s_{bi}(t)$ of the fuze ground echo can be obtained as follows:

(6) $$s_{b}(t)=\sum_{i=1}^{N_{u}}\sqrt{\frac{{P_{t}}^{2}{G_{i}}^{2}\lambda^{2}\sigma_{i}}{{\left(4\pi \right)}^{3}R_{i}{\left(t\right)}^{4}}}\cos\left\{2\pi \left[\frac{1}{2}\beta {\left(\frac{2R_{i}(t)}{c}\right)}^{2}\left.\left.-f_{0}\frac{2R_{i}(t)}{c}-\beta \frac{2R_{i}(t)}{c}t\right]+\varphi_{0}\right\}\right.\right.$$

In Equations (5) and (6), where $P_{t}$ represents the power of the transmitted signal, $G_{i}$ denotes the antenna gain, λ is the operating wavelength of the fuze, $R_{i}$ represents the distance, $\sigma_{i}$ is the equivalent radar cross-section, $\phi_{0}$ denotes the initial phase, and $N_{u}$ is the total number of scattering units within the ground echo region. In the following, the analysis of the altimetry algorithm will use the simulated beating signal of the fuzed ground echo given by Equations (5) and (6).

### 2.2. Extended Characteristics of Ground Echo Signals

From a geometric perspective, the scattering units within the ground echo region that share the same distance from the fuze form an equal distance echo region. This region corresponds to a segment of a circular arc, which is the intersection of a cone—whose apex is at the fuze antenna and whose central axis is perpendicular to the ground—with the ground surface. This arc is referred to as the equal distance arc. Similarly, the scattering units within the ground echo region that share the same Doppler shift constitute an equal Doppler echo region. This region corresponds to a segment of an elliptical arc, which is formed by the intersection of a cone—centered along the fuze’s velocity vector—with the ground surface. This arc is referred to as the equal Doppler elliptical arc.

**Figure 2:**
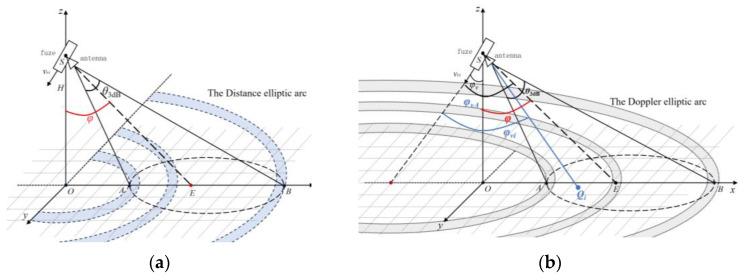
Geometric model of the fuze ground echo region: (**a**) the rule of distance extension of the ground echo region and (**b**) the Doppler spreading rule of the ground echo region.

As can be seen in Figure 2a, the closest scattering unit to the fuze is denoted as point A, while the farthest scattering unit to the fuze is denoted as point B. The distance between all scattering units on the ground and the fuze are bounded by SA and SB, satisfying the condition SA ≤ R ≤ SB. If the ground incidence angle of the fuze antenna beam center direction is φ, the angle of the half-power beam of the fuze antenna is $\theta_{3dB}$ and the height of the fuze from the ground at this moment is *H*, then the magnitudes of $r_{SA}$ and $r_{SB}$ are respectively given by

(7) $$r_{SA}=\frac{H}{\cos\left(\varphi -\frac{1}{2}\theta_{3\mathrm{dB}}\right)}$$

(8) $$r_{SB}=\frac{H}{\cos\left(\varphi +\frac{1}{2}\theta_{3\mathrm{dB}}\right)}$$

According to Equation (1), with the ranging formula for the beating frequency in the sawtooth wave FM fuze, ignoring the Doppler effect, it can be derived that the frequency range of the beating signal generated by all scattering units within the ground echo region is as follows:

(9) $$f_{b}\in \left[\beta \frac{2\times r_{SA}}{c},\beta \frac{2\times r_{SB}}{c}\right]$$

Therefore, when the fuze detects the ground, the beat frequency of the fuze echo undergoes a frequency expansion phenomenon.

As can be seen in Figure 2b, among the vectors from the fuze to each scattering unit, the vector $\vec{SA}$ from the fuze to the scattering unit *A* has the smallest angle with the fuze velocity $\vec{v}$, while the vector $\vec{SB}$ from the fuze to the scattering unit *B* has the largest angle with the fuze velocity $\vec{v}$. If the ground incidence angle of the fuze velocity is $\varphi_{v}$, then the relative velocity range between the fuze and each scattering unit within the echo region is as follows:

(10) $$v_{ri}\in \left[v\cos(\varphi_{v}+\varphi +\frac{1}{2}\theta_{3\mathrm{dB}}),v\cos(\varphi_{v}+\varphi -\frac{1}{2}\theta_{3\mathrm{dB}})\right]$$

If the angle between the fuze velocity and the antenna beam center is $\varphi_{vA}=\varphi_{v}+\varphi$, then the range of Doppler shift generated within the echo region is as follows:

(11) $$f_{di}\in \left[\frac{2v\cos(\varphi_{vA}+\theta_{3\mathrm{dB}}/2)}{\lambda },\frac{2v\cos(\varphi_{vA}-\theta_{3\mathrm{dB}}/2)}{\lambda }\right]$$

Therefore, when the fuze performs ground detection, the Doppler frequency components of the beating signal in the fuze echo will exhibit expansion.

Considering that the distance resolution and Doppler resolution of the fuze are not infinitely small, it can be assumed that the Doppler shift generated by scattering units at the same distance from the fuze is approximately identical. The Doppler shift of each scattering unit is a function of its distance from the fuze. By jointly solving the equations for the distance and Doppler shift of the scattering units relative to the fuze, it can be concluded that the distance and Doppler dimensions exhibit a joint expansion characteristic.

(12) $$f_{di}=\frac{2v}{\lambda }\cos\left[\arccos(\frac{H}{r_{i}})+\varphi_{v}\right]$$

When the ground incidence angle of the fuze velocity $\varphi_{v}$ is small, the Doppler shift $f_{di}$ is approximately inversely proportional to the distance $r_{i}$. This characteristic is referred to as the distance-Doppler joint extension characteristic of the fuze ground echo, and Equation (14) is called the characteristic function of the distance-Doppler joint extension.

## 3. Altimetry Algorithm in the Distance Dimension Based on the Improved CFAR with Secondary Protection Windows

Set $\frac{r_{SA}}{r_{SB}}=\frac{a}{b}$ and the ground incidence angle calculation formula can be obtained as follows:

(13) $$\tan(\varphi )=\frac{b-a}{b+a}\cot(\frac{\theta_{3\mathrm{dB}}}{2})$$

By substituting *φ* into Equation (7) or Equation (8), the altitude height above the ground of the fuze can be determined. Therefore, by simply measuring the minimum and maximum slant distances, i.e., obtaining the minimum and maximum frequency components of the frequency-modulated fuze’s beating signal, the impact angle estimation and accurate altimetry of the fuze can be achieved.

### 3.1. Design of the Altimetry Algorithm in the Distance Dimension Based on Improved CFAR

**Figure 3:**
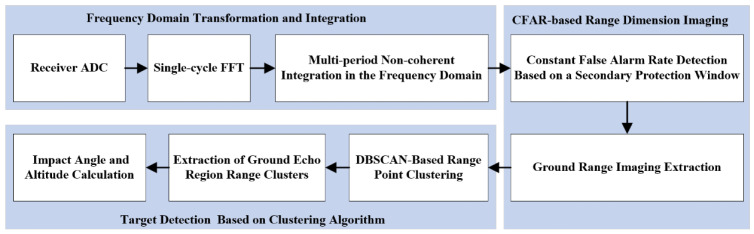
Fuze altimetry algorithm flow based on distance expansion characteristics.

The accurate altimetry algorithm for fuzes based on the distance extension characteristic contains four steps, and the flow of the algorithm is shown in Figure 3.

Step 1: Frequency domain transform and accumulation. First, the signal is transformed from the time domain to the frequency domain using the Fast Fourier Transform (FFT). Then, incoherent accumulation is applied in the frequency domain to enhance the SNR of the useful target signal.

Step 2: An improved CFAR algorithm with secondary protection windows is used for target detection of the accumulated signal to obtain the ground distance dimension imaging. And further refinement of target detection is required.

Step 3: Distance image target detection using the clustering algorithm. The Density-Based Spatial Clustering of Applications with Noise (DBSCAN) algorithm is applied to perform clustering analysis. This method identifies useful distance point clusters while eliminating interference signals and noise points, ultimately determining the maximum and minimum slant distances.

Step 4: Impact angle and altitude calculation. According to Equation (13), the impact angle of the fuze is determined from the maximum and minimum slant distances. Subsequently, Equation (7) is applied to obtain a more accurate altitude measurement.

### 3.2. An Improved CFAR Algorithm Based on Secondary Protection Windows

In this paper, the spectrum of the FM fuze beating signal is detected using the CFAR, and the target distance image is extracted. However, in the case of the traditional CFAR, the shielding effect between adjacent target points can lead to missed detections and inaccurate target extraction.

**Figure 4:**
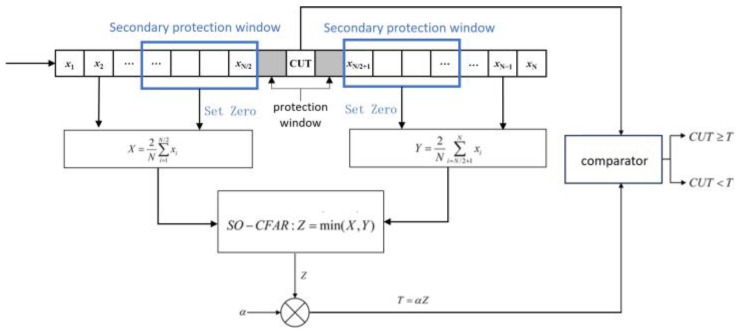
Improved CFAR algorithm based on secondary protection windows.

To address this issue, this paper proposes an improved CFAR detection algorithm based on secondary guard windows for extended distance targets, as illustrated in Figure 4. In this method, a secondary protection window is introduced within the adjacent range of the protection units. The length of the secondary protection window matches the total number of useful target sample points, and the sample points within the secondary protection window must be included in the reference window. When calculating the clutter power in the reference window, the amplitude of the sample points in the secondary protection window is first set to zero before computing the mean values X and Y in the reference window. SO-CFAR are min-based constant false alarm rate detection methods with low algorithmic complexity and good real-time performance. These methods are well-suited for radio fuze applications where computational resources are limited and real-time processing is required. The secondary protection window can protect the cell under test (CUT) threshold from being affected by adjacent sample points, ensuring that the threshold is determined solely based on the clutter power. By setting the length of the secondary guard window to the estimated total number of target sample points, the detection of edge sample points in extended range targets can be maximized, effectively improving target extraction accuracy.

**Figure 5:**
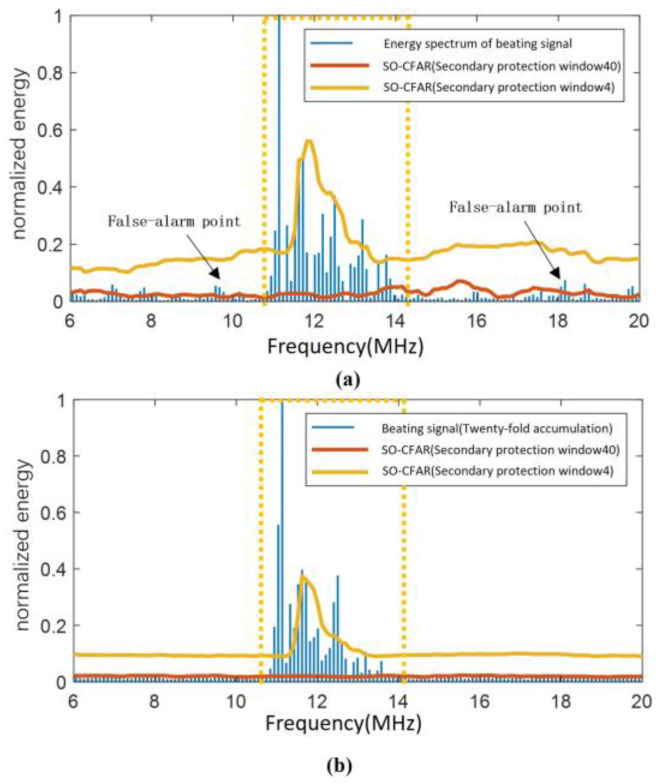
Comparison of CFAR detection of the beat frequency signal of the distance-extended target: (**a**) SO-CFAR and (**b**) SO-CFAR accumulation.

The classical Ulaby model [26] was used to fit the grassland conditions with a large backscattering coefficient, and the results of SO-CFAR target detection using this algorithm on the beating signal of the fuze at a distance of 100 m from the ground, with an antenna 3 dB beamwidth of 20° and a ground incidence angle of the antenna beam center of *φ* = 34°, are shown in Figure 5, with the false alarm rate of Pfa = 10^−5^. Figure 5a is the target detection result of the algorithm on the spectrum of the beating signal of one cycle with a total signal-to-noise ratio of 0 dB, and Figure 5b shows the results of target detection on the spectrum of the initial beating signal after 20 cycles of noncoherent accumulation. The solid blue lines in the figure represent the energy spectrum of the beating signal, while the solid red lines indicate the CFAR detection threshold when the secondary protection window length is set to 40. At this length, the secondary protection window is approximately equal to the number of target signal samples. The solid yellow lines represent the CFAR detection threshold when the secondary protection window length is set to 4, which is significantly smaller than the number of target signal samples. The region enclosed by the yellow dashed box marks the useful signal area, which can be used to roughly estimate the quantity of target samples.

From Figure 5, the following observations can be made:

Comparing Figure 5a,b, it is evident that the amplitude of the signal in the noise region (outside the yellow dashed box) is reduced by noncoherent accumulation, which can effectively improve performance.

In Figure 5b, comparing the threshold curves when the secondary protection window length is set to 4 and 40, it was found that the window length of 4 results in a severe shielding effect on distance-extended targets during the CFAR detection process. As a result, it can only detect the central part of the beating signal. In contrast, when the window length is set to 40, the algorithm effectively detects distance-extended targets without shielding effects. This demonstrates that the improved CFAR algorithm with proper secondary protection windows can protect the CUT from the amplitude variations of adjacent samples near the target signal.

**Figure 6:**
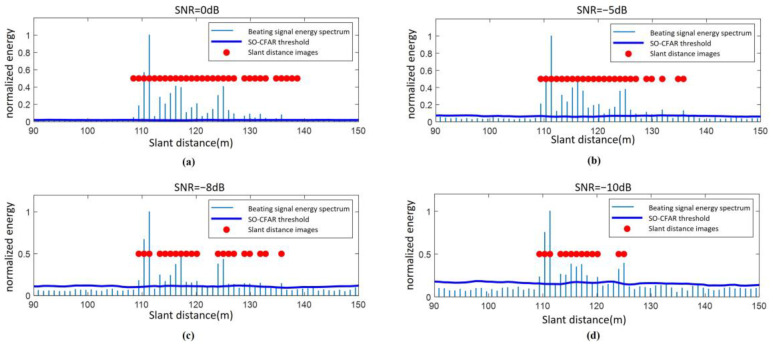
Roughness distance imaging based on the improved SO-CFAR: (**a**) SNR = 0 dB; (**b**) SNR = −5 dB; (**c**) SNR = −8 dB; (**d**) SNR = −10 dB.

The classical Ulaby model is employed to simulate wet snow-covered ground with a low backscattering coefficient. Under the same operating condition of the fuze, the SO-CFAR algorithm, incorporating secondary protection windows, is applied to detect the target from the fuze’s beating signal after incoherent accumulation. The distance points identified by SO-CFAR are extracted to generate the distance image of the ground under varying SNR conditions, as illustrated in Figure 6.

As shown in Figure 6 the proposed algorithm can still obtain a relatively complete ground range image even at the SNR of −8 dB. However, as the SNR decreases, the higher-frequency components of the beating signal gradually become submerged in noise. Consequently, points in the ground range image with larger slant distances (120 m to 135 m) begin to appear sparser, and noise-induced distance points emerge outside the useful distance points.

### 3.3. Distance Image Clustering Algorithm for Fuze Applications

In addition to the useful target distance, the ground distance image obtained through CFAR detection will contain some false alarm noise distance points and interference distance points. Therefore, further detection of the range image is necessary. In this paper, the DBSCAN clustering algorithm [27] is used to perform fine target detection on the distance image obtained through CFAR detection. The algorithm extracts the distance points in the ground echo region, removes interference and noise points, and generates a refined ground echo region distance image.

For a one-dimensional sequence of points, when the density threshold *MinPts* is defined as 2, the DBSCAN algorithm steps can be significantly simplified. As long as there is a point *q* within a distance *ε* in the positive or negative direction from point *p*, the point *p* is considered a core point, and the density reachability between point *p* and point *q* is established, with point *q* also being a core point.

Based on the above characteristics, the DBSCAN algorithm with a density threshold *MinPts* of 2 is used for target detection on the distance image sequence *D*(*d*) in one dimension. After the distance points are extracted using the secondary protection window-based CFAR detection algorithm, the points are arranged in ascending order to form the distance image sequence D(d),d∈[1,card(D)], where *card*(*D*) denotes the number of elements in the set *D*. The specific steps are as follows:

1. Define the neighborhood radius ε and initialize *d* = 1;
2. Create a new cluster *C* for the distance point *D(d)*;
3. If the distance between *D*(*d*) and *D*(*d + 1*) |*D*(*d + 1*) − *D(d*)| is less than the neighborhood radius *ε*, then the distance point *D*(*d*) and *D*(*d + 1*) belong to the same cluster *C*, and the other *d* = *d* + 1, skip to step 3.
4. If the distance between *D*(*d*) and *D*(*d + 1*) is greater than the neighborhood radius *ε*, the point *D*(*d*) is the last distance point in the cluster *C*. Skip to step 2 to create a new cluster for the point *D*(*d + 1*);
5. Repeat steps 2 to 4 until all points are traversed;
6. The number of distance points within each cluster is counted and the cluster with the highest number of distance points is considered as the target cluster.

**Figure 7:**
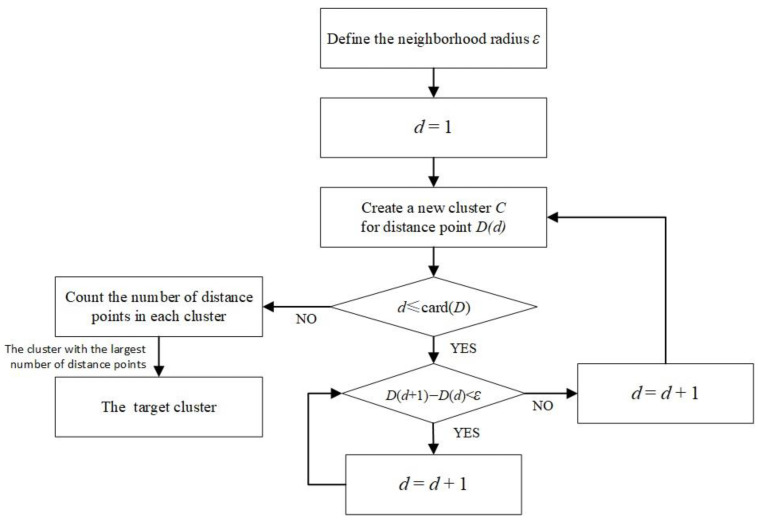
Ground distance image target detection algorithm based on DBSCAN.

The flow chart of the ground distance image target detection algorithm based on DBSCAN algorithm can be obtained as shown in Figure 7.

As shown in the flowchart, the algorithm only needs to traverse from the minimum distance to the maximum distance in the distance image to complete target detection. The complexity of the algorithm is *O*(*n*), making it suitable for fuze detection.

**Figure 8:**
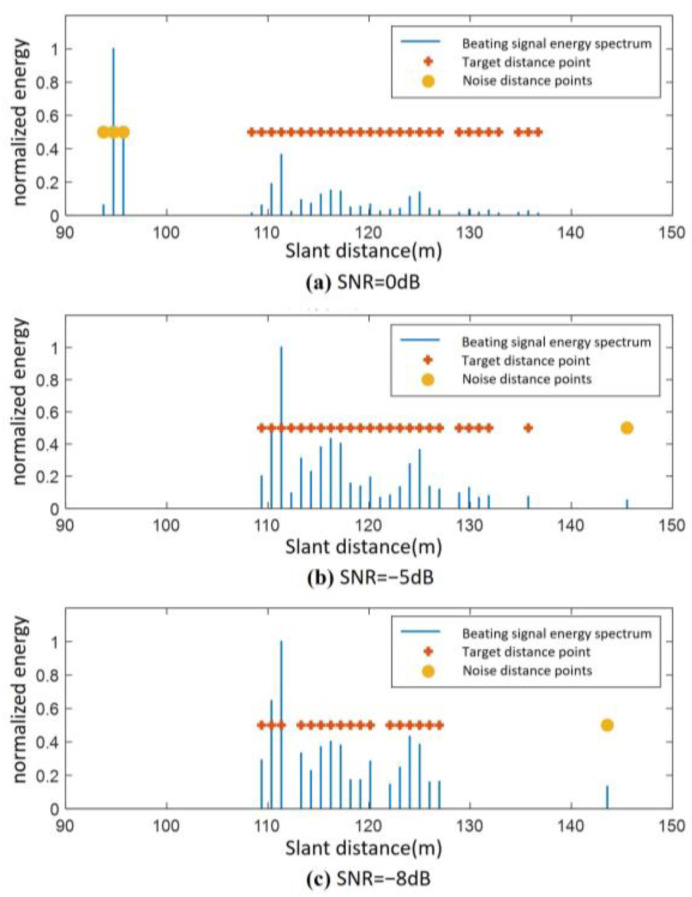
Distance image target detection results: (**a**) SNR = 0 dB; (**b**) SNR = −5 dB; (**c**) SNR = −8 dB.

By applying the above algorithm to the distance image shown in Figure 5, the target clustering results are shown in Figure 8. To verify the anti-jamming performance of the DBSCAN-based ground distance image target detection algorithm, a single-tone jamming signal is added at 95 m under a total SNR of 0 dB to simulate a forwarded jamming signal generated by a digital radio frequency memory (DRFM) jammer.

From Figure 8a, it can be seen that under the SNR of 0 dB, the algorithm successfully identifies the jamming signal at 95 m as a noise point. In Figure 8b,c, a false alarm noise distance is generated at the position of 143 m when the signal-to-noise ratio is −5 dB and −8 dB. After processing with the clustering algorithm, the false alarm noise distance is correctly determined as interference or noise.

## 4. Two-Dimensional Joint Altimetry Algorithm Based on Feature Region Fitting

In this section, the improved CFAR algorithm proposed in Section 3 is first utilized to estimate the range of slant distance, thereby reducing computational resource requirements. Subsequently, 2D joint altimetry is performed based on feature regions extracted from the 2D echo, enabling the accurate measurement of extended beat frequency.

The accuracy of altimetry based on the distance extension depends on the measurement accuracy of the maximum slant distance, while the frequency component of the beating signal of the maximum slant distance is susceptible to noise. Therefore, to ensure reliable altimetry performance of the fuze under low signal-to-noise ratio conditions and to enhance the adaptability of the high-flying fuzes to diverse geomorphological environments, a two-dimensional joint accurate altimetry algorithm based on the distance-Doppler joint extension characteristic is proposed. The proposed algorithm significantly improves altimetry accuracy compared to the one based solely on the distance extension characteristic. Additionally, it exhibits lower complexity than conventional two-dimensional signal processing algorithms, making it well-suited for implementation in fuzes with constrained resources and costs.

**Figure 9:**
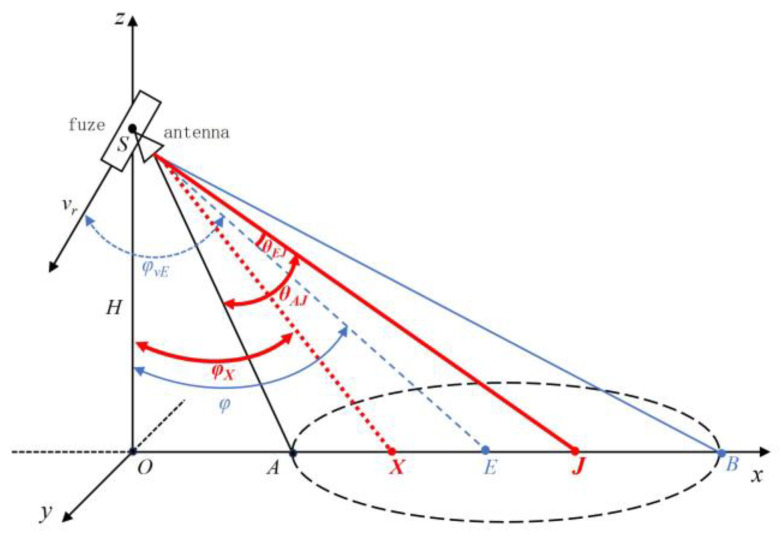
Geometric principle diagram of 2D joint accurate altimetry.

The geometric principle diagram of 2D joint accurate altimetry is shown in Figure 9. When the frequency component of the beating signal at the maximum slant distance $r_{SB}$ within the antenna irradiation area is submerged in noise, let the maximum detectable frequency component of the algorithm correspond to the slant distance of $r_{SJ}$, where $r_{SJ}$ is smaller than $r_{SB}$. The angle between the slant distances *SJ* and *SA* is denoted as $\theta_{AJ}$. If the altimetry algorithm based on the distance extension characteristic is used, an error exists in the impact angle and altimetry result because $\theta_{AJ}$ is smaller than $\theta_{3dB}$. To solve this problem, $\theta_{AJ}$ is determined using the velocities of point *A* and point *J* on the ground relative to the fuze, denoted as $v_{SA}$ and $v_{SJ}$. Then, $\varphi_{x}$ is derived using Equation (13), and finally, the accurate value of φ is calculated using $\varphi_{x}$.

Let the angle between *SJ* and the center of the fuze beam *SE* be $\theta_{EJ}$, then the velocity of point *J* on the ground relative to the fuze, denoted as $v_{SJ}$, can be obtained:

(14) $$v_{SJ}=v_{r}\cdot \cos\left(\varphi_{vA}+\theta_{EJ}\right)$$

Assuming that the fuze can always accurately measure the minimum slant distance *SA*, the velocity of point *A* relative to the fuze, denoted as $v_{SA}$, can be easily obtained:

(15) $$v_{\mathrm{SA}}=v_{r}\cdot \cos\left(\varphi_{vA}-\theta_{3\mathrm{dB}}/2\right)$$

By solving Equations (14) and (15) simultaneously, the angle $\theta_{AJ}$ between *SJ* and *SA* can be obtained as:

(16) $$\theta_{AJ}=\theta_{EJ}+\frac{1}{2}\theta_{3dB}=\arccos\left(\frac{v_{SJ}}{v_{r}}\right)-\arccos\left(\frac{v_{SA}}{v_{r}}\right)$$

In this paper, the angle $\theta_{AJ}$ is referred to as the detectable 3 dB beamwidth of the fuze antenna. According to Equation (13), $\varphi_{X}$ can be obtained:

(17) $$\varphi_{X}=\arctan\left[\frac{r_{SJ}-r_{SA}}{r_{SJ}+r_{SA}}\cot(\frac{\theta_{AJ}}{2})\right]$$

From $\varphi_{X}$, a more accurate value of φ can be obtained as $\varphi =\varphi_{X}+(\theta_{3\mathrm{dB}}-\theta_{AJ})/2$. Substituting φ into Equation (7) yields the height of the fuze relative to the ground.

### 4.1. Design of the 2D Joint Altimetry Algorithm Based on Feature Region Fitting

**Figure 10:**
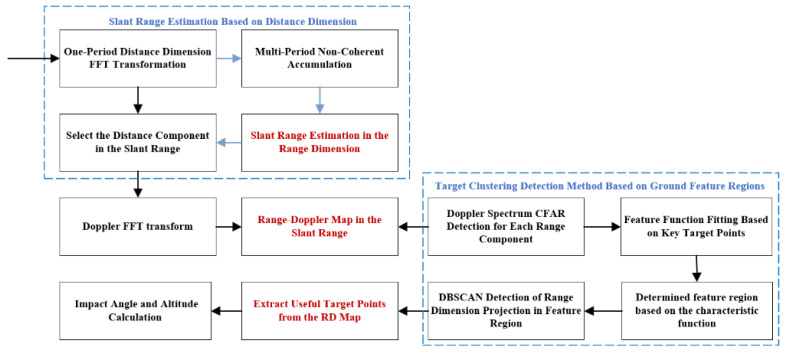
Two-dimensional joint accurate altimetry algorithm design.

The 2D joint accurate altimetry algorithm contains the following steps (shown in Figure 10):

Step 1: The slant distance $\left[R_{\min-r},R_{\max-r}\right]$ is preliminarily estimated according to the range-dimension imaging and target detection algorithm based on the improved CFAR described in Section 3.

Step 2: Doppler-dimensional FFT and CFAR detection. The distance components within the $\left[R_{\min-r}-\Delta R/2,R_{\max-r}+\Delta R\right]$ region are selected and subjected to the Doppler FFT. Subsequently, Doppler-dimensional target detection is performed on each distance-dimensional component in the range-Doppler (RD) map using constant false alarm detection to obtain the results of the distance-Doppler two-dimensional distribution of the ground echoes.

Step 3: Eigenfunction fitting and feature region determination. *K* key target points with relatively high amplitude are selected from the RD distribution of the ground echo. A first-order polynomial fitting is performed to obtain the joint distance-Doppler extension pattern of the ground echo $v=k_{1}r+k_{2}$ (eigenfunction). Then, we determine the useful target region (feature region) $T\left\{(r,v)|v=k_{1}r+k_{2}+k_{3}\right\}$, $r\in \left[R_{\min-r}-\Delta R/2,R_{\max-r}+\Delta R\right]$, $\;k_{3}\in [-\Delta V,\Delta V]$ in the RD distribution map.

Step 4: Clustering algorithm for accurate detection. The target points within the feature region T are projected onto the distance dimension. The DBSCAN algorithm is then applied for target detection within the distance dimension to obtain the beating signal as well as the Doppler frequency corresponding to the maximum and minimum slant distance.

Step 5: Fuze impact angle and height measurement. Based on the beating signal and Doppler frequency corresponding to the maximum and minimum slant distance, the value of the maximum and minimum slant distance and the value of the velocity component are calculated. According to Equations (7) and (17), the impact angle and height of the fuze are obtained.

### 4.2. Estimation of the Range of Slant Distance with Reduced Computational Resources

Performing Doppler-dimensional FFT sequentially on all distance components in the distance-dimension FFT matrix requires a large number of FFT operations. To address this, before performing the Doppler-dimensional FFT, the slant distance range $\left[R_{\min-r},R_{\max-r}\right]$ of the ground echo signal is first estimated using the proposed improved altimetry algorithm based on the distance extension characteristic. Distance-dimensional FFT components affected by interference and noise are then removed, and Doppler-dimensional FFT is applied only to the remaining components within the estimated range, significantly reducing the computational resources required for the two-dimensional FFT.

Since the altimetry algorithm in Section 3 is highly susceptible to noise, the estimated slant distance range is generally less than or equal to the true slant distance range. Therefore, it is necessary to extend the slant distance estimation range $\left[R_{\min-r},R_{\max-r}\right]$ after obtaining it. Given that the estimation error is smaller at a shorter slant distance and larger at a longer slant distance, the original estimated distance is adjusted by extending the minimum slant distance leftward (decreasing by ΔR/2, and the maximum slant distance rightward (increasing by ΔR). Finally, the extended slant distance range is $\left[R_{\min-r}-\Delta R/2,R_{\max-r}+\Delta R\right]$, where $\Delta R=R_{\min-r}-R_{\max-r}$.

**Figure 11:**
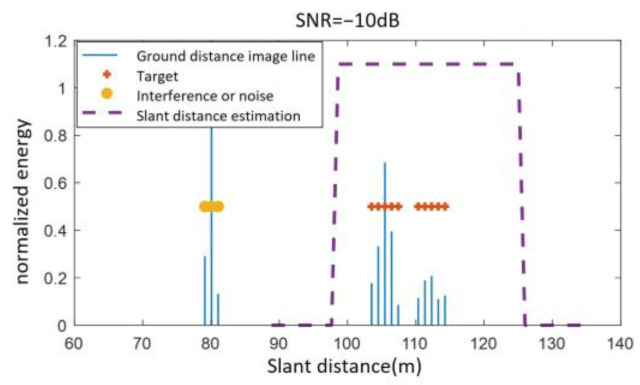
Slant distance estimation results at −10 dB SNR.

Given a fuze altitude of 100 m, a velocity of 500 m/s, an antenna beam center ground incidence angle of 25°, and an antenna 3 dB beamwidth of 20°, the theoretical slant distance range of the fuze to the ground echo region is [103.52 m, 122 m]. Under the condition that SNR = −10 dB, the slant distance is estimated using the improved altimetry method based on the distance extension characteristic. As shown in Figure 11, the estimated values are $R_{\min-r}$ = 103.5 m, $R_{\max-r}$ = 115.2 m, and the extended slant distance range is adjusted to [97 m, 127 m].

**Figure 12:**
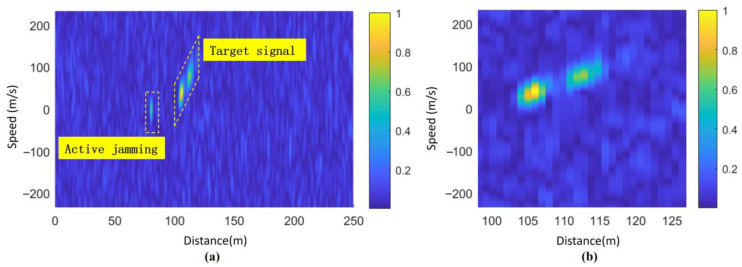
Beating signal range Doppler spectrum image: (**a**) full-range RD spectrum image and (**b**) estimated range RD spectrum image.

The RD image results of Doppler FFT in the entire range of the beating signal are shown in Figure 12a. Doppler FFT is performed on the beating signal after incoherent accumulation within the range of slant distance estimation, and the resulting RD map is shown in Figure 12b. It can be seen that Figure 12b is the section of Figure 12a in the range of (97 m,127 m), without any loss of information, while the interference signal located at (80 m, 0 m/s) is effectively eliminated.

### 4.3. Accurate Altimetry Based on Feature Region Extraction and Extended Beating Frequency from 2D Echoes

**Figure 13:**
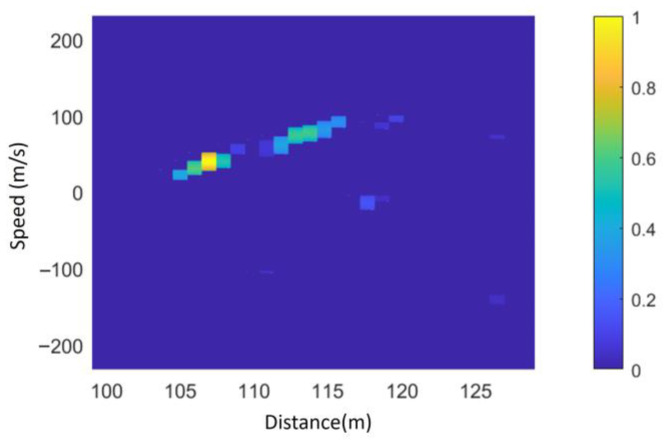
Doppler dimensional CFAR detection results.

According to the joint distance-Doppler extension characteristic of the ground echo, scattering units on the ground that share the same distance with the fuze produce identical Doppler shifts. Therefore, a CFAR is applied to detect the Doppler spectrum of each range component. By performing detection on the RD map in Figure 12a, the detected ground RD distribution is obtained, as shown in Figure 13.

As observed in Figure 13, the RD distribution of ground echoes still contains numerous noise points, making it difficult to directly extract the maximum and minimum slant distances and their corresponding velocities.

**Figure 14:**
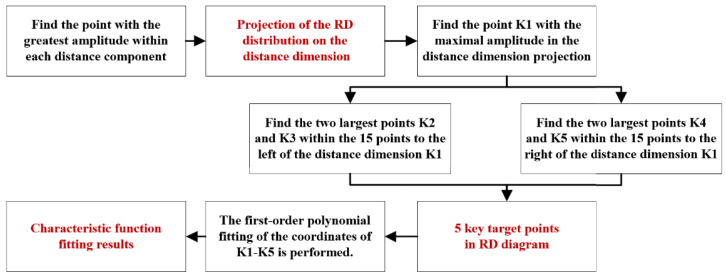
The algorithm flow of identifying key target points.

To address this, a useful target region is extracted from the echo RD distribution based on the distance-Doppler joint extension characteristic of ground echo signals, effectively removing noise points. In this study, the parameter of *K* is set to 5, and the algorithm flow for identifying key target points follows the process illustrated in Figure 14.

**Figure 15:**
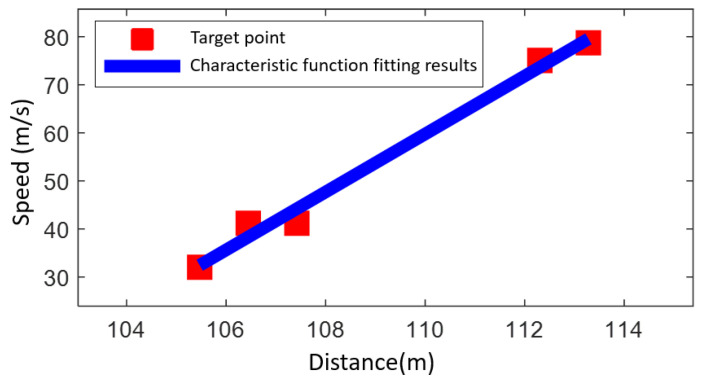
Results of the first order polynomial fitting of the eigenfunction.

In this paper, the fitting results are approximated as eigenfunctions of the joint distance-Doppler extension. Figure 15 shows the results of the first-order polynomial fitting of the eigenfunction.

**Figure 16:**
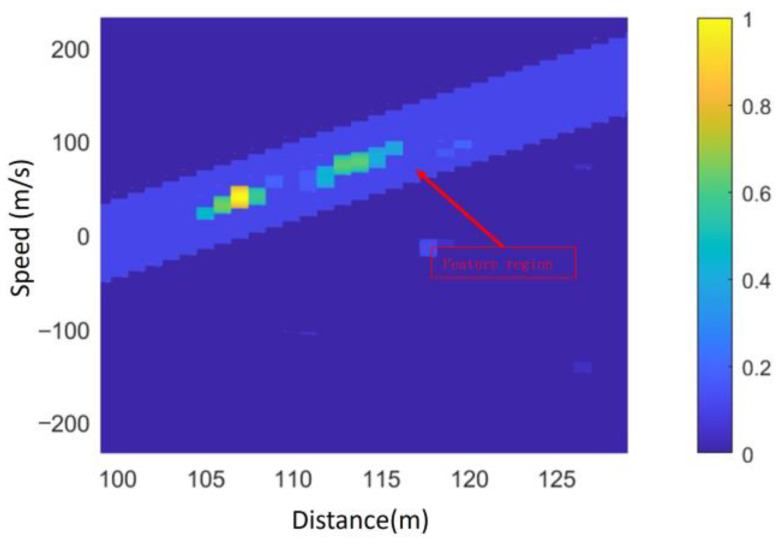
Results of feature region division.

Due to the inherent fitting error between the extracted range-Doppler joint extension characteristic function and Equation (12), useful targets cannot be directly selected based on the fitted eigenfunctions. Instead, the feature region is determined by selecting the fitted feature function within the range of ±ΔV in the Doppler dimension. As shown in Figure 16, the extracted feature region effectively retains the main target information while removing noise points outside the region.

A small number of residual noise points within the feature region are further processed using the DBSCAN-based clustering detection method described earlier. All target points in the feature region are projected onto the distance dimension, where target detection is performed to eliminate interference and noise points. This process ultimately yields the beating frequency $f_{bv}$ and Doppler frequencies $f_{d}$ corresponding to the maximum and minimum slant distances.

**Figure 17:**
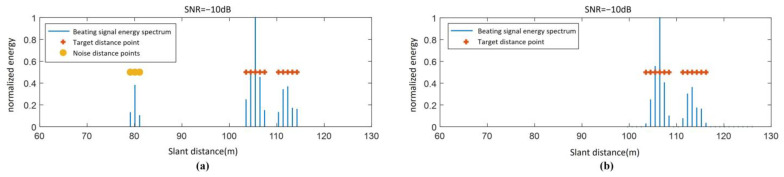
Algorithm detection results (SNR = −10 dB): (**a**) one-dimensional algorithm detection results and (**b**) distance dimension projection of RD distribution in the feature region.

The detection results using the proposed algorithm based on the joint extension characteristic in both distance and Doppler domains are shown in Figure 17b, while the results obtained using the conventional range extension-based detection algorithm are presented in Figure 17a. As observed in Figure 17 the detected range in Figure 17b spans [104 m, 117 m], whereas in Figure 17a, the detected range is [105 m, 115 m]. This demonstrates that the clustering detection algorithm based on the ground feature region proposed in this section exhibits superior detection capability.

Using the extracted range information, the velocities corresponding to the maximum and minimum slant distances are determined and substituted into Equation (16) to obtain the precise height of the fuze relative to the ground. Furthermore, since the proposed detection algorithm does not require traversing the entire two-dimensional RD map, it has significantly lower computational complexity compared to the conventional two-dimensional CFAR, making it more suitable for fuze target detection.

Additionally, the improved CFAR algorithm introduced in this study incorporates secondary protection windows, enhancing detection performance in complex environments. Moreover, the unique feature region extraction approach effectively reduces the computational burden by limiting the search space, further improving processing efficiency.

## 5. Comparison of Simulation Results

### 5.1. Simulation Analysis of Altimetry Results in the Distance Dimension

**Figure 18:**
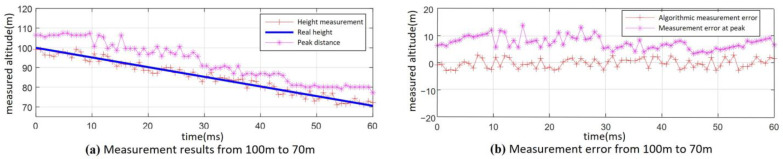
Fuze from 100 m to 70 m in the whole-process measurement results (SNR = 0 dB): (**a**) measurement results and (**b**) measurement error.

**Figure 19:**
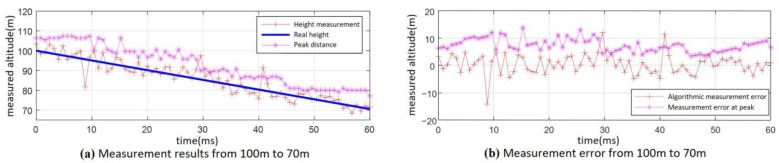
Fuze from 100 m to 70 m in the whole-process measurement results (SNR = −10 dB): (**a**) measurement results and (**b**) measurement error.

For the altimetry performance simulation in practical operations, the following parameters were set: the fuze antenna 3 dB beamwidth was 20°, the ground incidence angle of the antenna beam center was *φ* = 34°, the fuze velocity ground incidence angle was 2°, and the velocity was 500 m/s. The height measurement algorithm proposed earlier was applied to process the fuze’s beating frequency signal during its descent from 100 m to 70 m under both 0 dB and −10 dB SNR conditions. The data were processed every 15 modulation cycles. The resulting height measurement performance over the entire process is shown in Figure 18 and Figure 19.

The altimetry results for the fuze’s descent from 100 m to 70 m under the condition of 0 dB SNR are shown in Figure 18. The results of the altimetry algorithm based on distance dimension extension characteristics are significantly better than those obtained with the peak detection method. It can be determined through calculation that the maximum error of using the peak value method is 14.6 m, and the overall height measurement RMSE is 9.48 m. The error ratio is approximately 9.5%. In contrast, the algorithm presented in this paper achieves a maximum error of 3.6 m, with an overall height measurement RMSE of 1.77 m. The error ratio is approximately 1.8%.

As can be seen from Figure 19, under the SNR of −10 dB, the height measurement results show more significant fluctuations throughout the entire process compared with Figure 18. However, the height measurement results obtained using the algorithm proposed in this paper still outperform those from the peak detection method. Despite this, two large measurement errors occurred at 91 m and 54 m, respectively. As a result, the maximum error is 15.3 m, and the whole-process height measurement RMSE is 5.13 m. The error ratio is approximately 5.2%.

Through the above simulation analysis, the accuracy of the altimetry algorithm based on distance dimension extension characteristics is better than the peak height measurement method when the SNR of the fuze’s beat frequency signal is greater than 0 dB. The RMSE of height measurement under different impact angles, heights, and ground conditions does not exceed 3 m. However, when the SNR is small, such as below 0 dB, the higher frequency components in the beat frequency signal are gradually submerged by noise, causing the points at larger slant distance to become sparse. This leads to instability in the height measurement performance during the fuze’s descent process.

### 5.2. Simulation Analysis of the 2D Joint Accurate Altimetry Algorithm

As analyzed in the previous sections, traditional distance-dimension processing methods for altimetry often suffer from limited accuracy due to their inability to effectively utilize the joint distance-Doppler characteristics of ground echoes. These methods are particularly prone to errors under low SNR conditions, leading to reduced reliability in complex environments. To address these limitations, this study employs the proposed two-dimensional joint accurate altimetry algorithm, which integrates both distance and Doppler domain features for enhanced performance.

**Figure 20:**
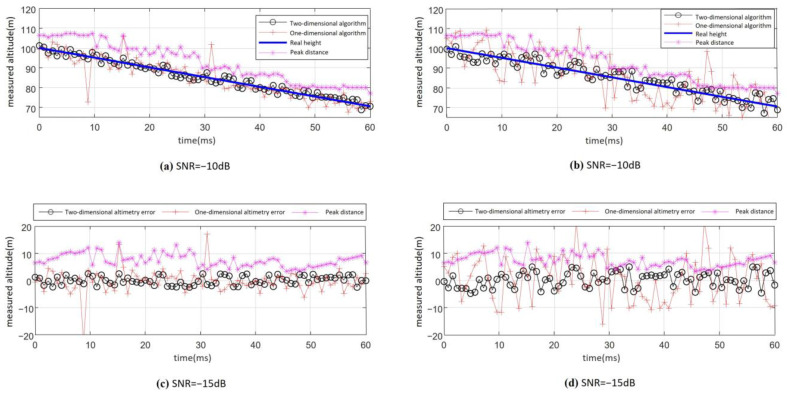
Measurement results and error from 100 m to 70 m: (**a**) measurement results (SND = −10 dB); (**b**) measurement error (SND = −10 dB); (**c**) measurement results (SND = −15 dB); (**d**) measurement error (SND = −15 dB).

Set the fuze antenna 3 dB beam width to 20°, the antenna beam center ground incidence angle φ=34°, and the fuze velocity to 500 m/s. The proposed altimetry algorithm is applied to process the beat frequency signal with a −10 dB SNR as the fuze’s height decreases from 100 m to 70 m. Processing is performed every 20 frequency modulation cycles, and the complete height measurement results are shown in Figure 20a,b. As can be seen from the figure, the altimetry performance throughout the entire process is highly stable, with a maximum error of 3.1 m. Compared to the one-dimensional algorithm (altimetry based on distance extension), there is a significant improvement in performance. As a result, the proposed 2D joint altimetry algorithm’s RMSE is 2.38 m and the error ratio is approximately 3%.

The whole-process altimetry results obtained from the beating signal with SNR of −15 dB under the same condition are shown in Figure 20c,d. As observed from the figure, the two-dimensional joint altimetry algorithm still maintains relatively stable performance at an SNR of −15 dB. Throughout the entire process, no abnormal height points caused by algorithm failure were observed. As a result, the RMSE of the whole-process altimetry is 4.76 m and the error ratio is approximately 5%.

Through the above simulation analysis, the proposed 2D joint accurate altimetry algorithm demonstrates significant performance improvement compared to the distance-extension-based altimetry algorithm. It achieves more accurate and stable height estimation even under low SNR conditions. In particular, when the SNR is greater than −10 dB, the RMSE of height measurement is less than 3 m. The error ratio is approximately 3%. When the SNR is −15 dB, the measurement performance is still stable, with the height measurement RMSE of less than 5 m and the error ratio of approximately 5%.

To preliminarily verify the effectiveness of the proposed method, a performance comparison was conducted among several approaches. However, research on fuzes with burst heights exceeding 60 m remains limited. Therefore, the latest fuze ranging algorithms developed in the past five years for burst heights within the 0–50 m range were selected for comparison [28,29,30,31,32]. The comparison results are shown in Table 1.

From the comparison in Table 1, it can be observed that Algorithm 2 exhibits a greater ranging error than the proposed method, while Algorithms 1 and 5 also show higher ranging errors under the same or even better SNR conditions. Although Algorithm 3 does not explicitly specify the SNR conditions, its ranging error at 15 m exceeds that of the proposed method at 70 m under −10 dB SNR. Furthermore, Algorithm 4 appears to have a lower ranging error, but its test conditions involve a significantly higher SNR than those considered in this study.

It is important to note that as the detection distance increases, the signal undergoes greater path loss during propagation, leading to an exponential decline in SNR and a significant increase in signal processing complexity. Despite these challenges, the proposed 2D joint altimetry algorithm maintains a ranging error of no more than 5% at extended detection distances (70–100 m) and under low SNR conditions (−10 dB/−15 dB). This demonstrates the superior detection capability and robustness of the proposed method, proving its practical applicability in altimetry tasks in challenging operational environments.

## 6. Measured Performance Verification

**Figure 21:**
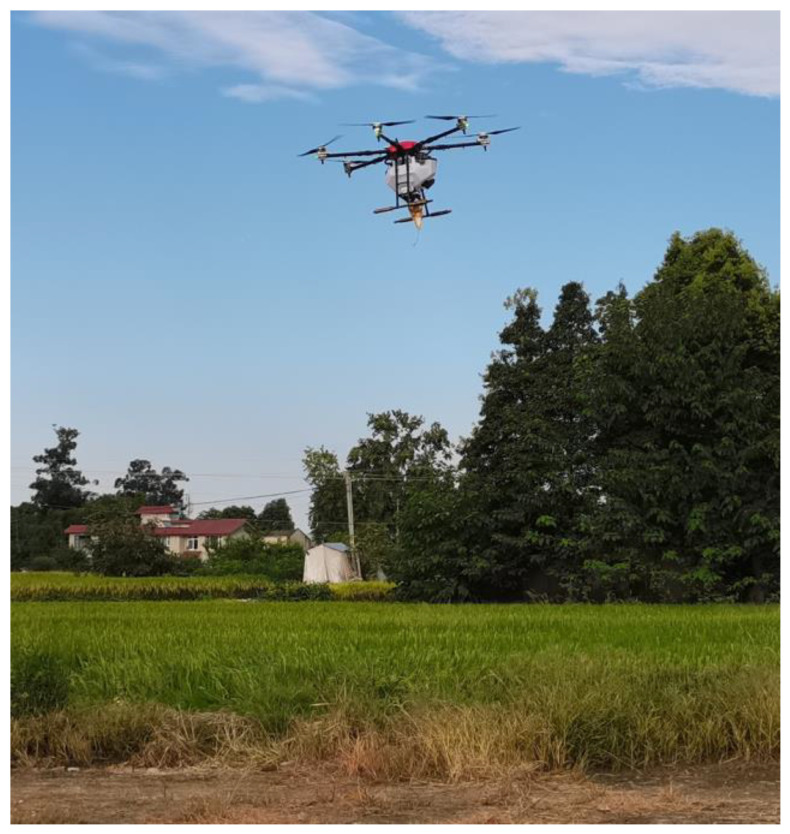
Experimental test setup for the fuze prototype.

In this study, to evaluate the performance of the fuze prototype in real-world operation, an LED strip was connected to the ignition signal. The activation time of the fuze was determined based on the moment when the LED transitioned from dark to bright. To verify the burst height accuracy of the height-of-burst fuze in actual high-altitude conditions, the fuze prototype was mounted on a drone, which descended vertically from 140 m to 60 m. The altitude at which the LED switched from dark to bright was observed and recorded to determine the activation height of the fuze. The experimental setup for the drone-mounted flight test of the fuze prototype is shown in Figure 21.

The recorded detonation height results at 100 m and 80 m under a fuze angle of 35° are presented in Table 2. Analysis of the experimental data indicates that the maximum height measurement error at 100 m is 6.2 m, with a root mean square error (RMSE) of 5.03 m. At 80 m, the maximum height measurement error is 5.0 m, with an RMSE of 3.73 m. The error remains within 5.5%.

## 7. Conclusions

This study investigates the detection method of high-altitude airburst fuzes, achieving accurate altimetry under narrow-beam antennas. First, the distance extension and Doppler extension characteristics of fuze ground echoes were theoretically analyzed. Based on this, a two-dimensional joint extension characteristic fuze altimetry algorithm was proposed, incorporating an improved CFAR detection and an accurate feature region extraction method. The main contributions of this study are as follows:

1. An improved CFAR detection method based on secondary protection windows was proposed, effectively mitigating the masking effect caused by the classical CFAR algorithm. By leveraging the geometric relationship between maximum and minimum slant distances and the impact angle, the ground-referenced altimetry of the fuze was achieved in the distance dimension;
2. By integrating Doppler extension characteristics into the single-dimensional distance-based algorithm, a two-dimensional joint accurate altimetry algorithm was developed, significantly improving height measurement accuracy under low SNR conditions. Furthermore, the proposed feature region extraction method was employed to refine the estimated impact angle;

The simulation results validate the effectiveness and robustness of the proposed algorithm for fuzes operating at burst altitudes ranging from 70 m to 100 m. Under an SNR of −10 dB, the RMSE is less than 2.38 m, with an error rate of approximately 3%. Notably, even under extremely low SNR conditions of −15 dB, the RMSE remains below 4.76 m, with an error rate not exceeding 5%. Additionally, real-world measurements confirm that for fuzes maintaining a fixed altitude of 80 m and 100 m, the RMSE does not exceed 5.5 m, with an error rate of no more than 5.5%. These results demonstrate that the proposed method achieves higher accuracy and stability compared to conventional approaches, particularly in low-SNR scenarios.

## Figures and Tables

**Table 1:** Performance comparison of different altimetry algorithms.

	Altimetry Algorithm	SNR (dB)	Ranging Distance (m)	Ranging Error (m)	Error
1	Ranging method of hybrid modulation fuze based on instant correlation frequency domain detection	−10	6	0.25	4.1%
2	High-accuracy ranging method of an FMCW detector based on Fourier coefficient interpolation	−10	5–10	0.76	7.6%
3	Simulation Experiment of a Fixed-Distance Optimization Algorithm	Not mentioned	15	0.567	3.64%
4	Multi-carrier Phase Ranging Algorithm Based on OFDM Technology	15	5–30	0.5	1.7%
5	Ranging Method for Linear Frequency Modulation radio Fuze Based on Fractional Fourier Transform	0	0–50	2	4%
6	Proposed 2D Joint Altimetry Method	−10	70–100	2.38	2.38%
		−15		4.76	4.76%

**Table 2:** Recorded detonation height results of the fuze at 35° on bare soil.

	Parameter	Index										RMSE (m)	Error Ratio
80 m Test	Trial Number	1	2	3	4	5	6	7	8	9	10	3.73	4.6%
	Activation Height (m)	83.9	83.9	81.7	75.3	85.0	76.3	79.2	84.4	77.6	75.2		
100 m Test	Trial Number	1	2	3	4	5	6	7	8	9	10	5.03	5.1%
	Activation Height (m)	95.7	95.4	95.8	94.4	95.1	105.9	106.2	103.0	95.0	94.1		

## Data Availability

The data presented in this study are available on request from the corresponding author. The data are not publicly available due to confidentiality and security restrictions.
